# The immunological landscape of traumatic brain injury: insights from pathophysiology to experimental models

**DOI:** 10.3389/fneur.2025.1668480

**Published:** 2025-09-18

**Authors:** Matthew Abikenari, Joseph H. Ha, Justin Liu, Alexander Ren, Kwang Bog Cho, Jaejoon Lim, Lily H. Kim, Ravi Medikonda, John Choi, Michael Lim

**Affiliations:** ^1^Department of Neurosurgery, Stanford University School of Medicine, Stanford, CA, United States; ^2^Nuffield Department of Clinical Neurosciences, University of Oxford, Oxford, United Kingdom

**Keywords:** traumatic brain injury (TBI), cerebrovascular immunology, immunopathology, controlled cortical impact (CCI), fluid percussion injury (FPI)

## Abstract

Traumatic brain injury (TBI) is a complex, heterogeneous neuropathological disease that continues to be among the prominent causes of mortality and disability around the world. Translational success in TBI has been significant, yet therapies are limited as the intersection of the initial mechanical traumas and secondary neuroinflammatory cascades, which predispose to long-term neurological deficits, is poorly understood. The pathogenesis of TBI is not limited to the primary mechanical injury. The secondary damage, including ischemia, excitotoxicity, oxidative stress, and immune dysfunction, leads to neuronal apoptosis, the breakdown of the blood–brain barrier (BBB), and chronic neuroinflammation. The preclinical controlled cortical impact (CCI) and fluid percussion injury (FPI) TBI models have generated valuable biomechanical data related to TBI-induced immune responses, including microglial priming, astrocyte dysregulation, and peripheral leukocyte recruitment. However, experimental models today are unable to completely replicate the intricate immune cascades in human TBI, particularly delayed and context-specific innate and adaptive immune response activation. Cytokine signaling (IL-1β, TNF-*α*, and IL-6), neuroinflammatory amplification through the IL-23/IL-17 pathway, and autoantibody-mediated neurodegeneration are emerging as significant secondary injury mechanisms. Additionally, TBI-induced immunosuppression, which presents as generalized T lymphocyte depletion and aberrant macrophage polarization, enhances the risk of infection and delayed neurological recovery. Emerging immunotherapeutics such as cytokine blockade, complement blockade, and targeted modulation of T lymphocytes have the potential to optimize the post-TBI immune microenvironment for reducing secondary damage. Inclusion of next-generation experimental models combined with secondary injuries, such as hypoxia, polytrauma, and systemic inflammation, is needed to shift towards innovative, biomarker-driven, patient-stratified trials. Thus, integration of immunological phenotyping with translationally relevant models of TBI represents an important cornerstone in the development of targeted therapeutic treatments designed to improve neuroprotection, repair, and long-term functional outcome.

## Introduction

Traumatic brain injury (TBI) is the leading cause of mortality and morbidity in individuals under the age of 45. This results in substantial economic and societal burdens due to lost productivity and long-term disability (1). Despite contemporary advancements in understanding the pathophysiology of intracranial injuries, the ability to precisely reconstruct the sequence of events leading to trauma and accurately predict injury severity and progression remains a significant challenge (2, 3). The clinical complexity of TBI stems from its heterogeneous nature, involving a dynamic interplay between mechanical damage, secondary biochemical cascades, and a dysregulated immune response. Despite advances in acute care, little is known about the pathophysiological mechanisms that govern long-term recovery and prognosis following traumatic brain injury. Thus, experimental models of TBI serve as a critical tool in elucidating such essential mechanisms, providing insights into neuroinflammatory processes, immune cell recruitment, and secondary injury pathways that shape post-traumatic recovery and therapeutic intervention. The current paper elucidates the immunological landscape of TBI and its integration with experimental models in order to identify potential therapeutic targets that can be translated into clinical practice.

TBI is defined as an injury to the brain caused by an external mechanical force, such as blast waves, crushing forces, impact injuries, projectile penetration, and abrupt acceleration-deceleration forces (3, 4). The proceeding injuries from any combination of the above forces will lead to focal brain damage due to contact phenomena or diffuse brain damage due to acceleration/deceleration inertial phenomenon. Focal brain damage can result in lacerations, contusions, and intracranial hemorrhages, while diffuse brain damage can result in brain swellings and diffuse axonal injuries. However, it is important to note that despite these classifications, TBI is not a single clinical phenomenon but a highly complex disease process with various structural impairments, dysregulated biochemical pathways, altered neuronal function, diminished regulations of cerebral blood flow (CBF), and dysregulated immune metabolism (5).

Neuropathological classification of such injuries is determined by primary and secondary injury insults. Primary insult results from the direct mechanical impact of the damage to the brain immediately following the accident, which can cause instantaneous axonal shearing and hemorrhage, and holds a very small window of therapeutic intervention. Secondary insult results from non-mechanical damage caused by cascades of dysregulated physiological, metabolic, and cellular proceedings that follow the primary insult (6, 7). Secondary insults can lead to cerebral swelling, hypertension, and diffuse and focal hypoxic–ischemic damage. Secondary insults are slow in their clinical manifestations and present a larger window for therapeutic intervention. Furthermore, the mechanism of insults resulting from secondary injuries includes alterations of key biochemical cascades such as homeostatic disturbances in cellular calcium and sodium channels, substantial glutamate excitotoxicity, mitochondrial damage, lipid peroxidation, neuroinflammation, increased generation of free radicals and increased concentration of intracellular free fatty acids, leading to eventual apoptosis and diffuse axonal injury (DAI) (4, 8, 9).

## Clinical pathophysiology and management of TBI

Traumatic Brain Injury (TBI) encompasses a spectrum of clinical severity, from mild concussion to profound coma, underpinned by complex neuropathological processes. Clinical presentation varies with the extent of initial mechanical trauma, with severe forms posing a dangerous risk of intracranial hypertension, hypoxemia, and late neurological sequelae. Clinical grading systems cannot, of themselves, explain pathophysiological variation affecting outcome (2, 3).

The trauma happens in two phases: the first insult is due to direct mechanical deformation of brain tissue, and the second phase, from hours to days due to metabolic breakdown, ischemia, excitotoxicity, and immune dysregulation. Ischemia produces lactic acidosis, oxidative stress, and ATP loss, disrupting ionic gradients and activating cascades of cell death. Among the characteristics of secondary injury is abnormal cerebral blood flow (CBF), triphasic in nature and involving hypoperfusion, hyperemia, and delayed hypoperfusion, each contributing in a characteristic fashion to tissue damage and worsening of clinical condition (3, 4).

Treatment of TBI is support-oriented and tiered by severity. Primary prevention, through safety equipment like helmets and seatbelts, is the sole truly effective intervention. Restriction of secondary insult is of urgent priority in the acute setting (4). This includes tight management of intracranial pressure (ICP), maintenance of cerebral perfusion pressure (CPP), and surgery to decompress when necessary. Pharmacologic treatment, such as NMDA-receptor antagonists, calcium channel blockers, and free-radical scavengers, has been studied but has not yet yielded consistent clinical benefit (8, 9). The inability of preclinical potential to be translated clinically emphasizes the need for improved experimental models that more accurately mirror the complex and temporally dynamic nature of human TBI. Table 1 recapitulates a comprehensive pathophysiological summary of traumatic brain injury.

**Table 1:** Comprehensive pathophysiological summary of traumatic brain injury (TBI).

Aspect	Description	Immunological component
Primary injury	Direct mechanical insult causing axonal damage, contusions, and hemorrhage (155).	Limited immune activation initially; rapid release of DAMPs (HMGB1, ATP, S100β) triggers innate immune responses, including microglial priming (156).
Secondary injury	Progressive biochemical cascades: ischemia, oxidative stress, excitotoxicity (157).	Neuroinflammation propagates via microglial activation, astrogliosis, and sustained cytokine release (IL-1β, TNF-α, IL-6), leading to prolonged BBB dysfunction (158).
Blood–brain barrier (BBB) disruption	Loss of BBB integrity due to endothelial and astrocytic damage (159).	Recruitment of peripheral immune cells neutrophils, monocytes, T cells through upregulated adhesion molecules (ICAM-1, VCAM-1) and chemokine gradients (CXCL1, MCP-1) (114, 160).
Neuroinflammation	Chronic activation of resident and peripheral immune cells (161).	Dysregulated M1/M2 microglial polarization influences recovery; M1 phenotype sustains injury via ROS and NO, while M2 phenotype promotes tissue repair (162, 163).
Oxidative stress	Excessive ROS, lipid peroxidation, mitochondrial dysfunction (164).	Microglial and neutrophil-derived ROS/NOS contribute to oxidative DNA damage and neuronal apoptosis; Nrf2 dysregulation exacerbates redox imbalance (165, 166).
Cerebral edema	Ionic dysregulation induces cytotoxic and vasogenic swelling (167).	IL-1β, TNF-α increase endothelial permeability; aquaporin-4 (AQP4) dysregulation on astrocytes contributes to cerebral fluid accumulation and edema progression (159, 168).

## Experimental models of traumatic brain injury

Given the heterogeneous pathophysiology of TBI in the patient population, numerous animal models have been developed over the last several decades that depict clinically relevant features of both focal and diffuse pathophysiologies. Although focal pathologies such as cerebral edema, hematomas, and contusions are well characterized in animal models, their translational value appears variable because clinical TBI often presents with diffuse rather than strictly focal injury patterns (10). Hence, animal models that focus on diffuse pathophysiologies with widespread impact, such as DAI, vascular injury and ischemia, may be more clinically applicable in certain contexts. This paradigm shift is also evident when examining the history of research in animal models of TBI. In contrast to early animal models of TBI that focused exclusively on the biochemical pathophysiology of focal impact injuries, contemporary models focus on the highly elongated molecular and cellular cascades that characterize secondary insult pathophysiology (5, 11).

There are currently four widely utilized animal models used in contemporary TBI research: controlled cortical impact (CCI) injury, fluid percussion injury (FPI), penetrating ballistic-like brain injury (PBBI) and weight-drop impact model. The CCI injury model uses an electromagnetic piston to drive and penetrate a rigid impactor onto exposed dura of known brain regions with a varying gradation of velocity to mimic cortical tissue loss with widespread axonal damage (12, 13). FPI model uses a fluid-filled piston to produce and subsequently inject a pressurized fluid pulse onto an intact dura to cause deformations of brain tissues, with varying degrees of severity, depending on the pulse strength (14, 15). PBBI model uses a projectile transmission of a metal rod with varying degrees of energy to cause a temporary cavity in the brain to induce widespread inflammation, cortical spreading depression and brain swelling (16–19). In weight-drop TBI models, an object of varying weight and height is dropped into gravitational free fall onto an exposed brain skull to cause severe cortical contusions and progressive hemorrhages (20–22). Although each model has unique experimental advantages and limitations in its ability to recapitulate a clinically relevant model of TBI, CCI and FPI tend to yield more consistent injury patterns and can be a useful model to simulate the immune responses seen in human TBI. Accordingly, this review focuses on the role of CCI and FPI models as preclinical therapeutic strategies for the treatment of TBI.

### Controlled cortical impact (CCI) injury and fluid percussion injury (FPI) models

The CCI model offers several practical strengths for translational applications. First, CCI can induce widespread diffuse degeneration of cortical and thalamic neurons, comatose states, and BBB dysfunction while controlling for crucial spatiotemporal parameters such as time, velocity and depth of injury across brain regions (14, 23–25). Furthermore, CCI models have been shown to induce cognitive deficits (Morris-water maze test) and emotional and behavioral impairments (forced swim test) that are well-preserved more than 12 months post-TBI injury (26–29). This model allows for the manipulation of velocity and depth of initial impact during the experiment, thereby controlling the severity of such pathophysiological, cognitive and emotional deficits (30, 31). Furthermore, increased gradations of impact velocity correspond to a progressive reduction in cerebral blood flow and elongated elevation of DAI and white matter atrophy. Hence, CCI models allow for collecting and extrapolating post-TBI physiological data in a context similar to ICU and intensive trauma centers. Because CCI reproduces several pathophysiological and behavioral features seen in human TBI, it may help connect preclinical and clinical work to translate animal models of TBI into novel protocols in clinical care (13, 27, 32, 33).

Furthermore, FPI may provide practical advantages in answering certain translational questions to study severe TBI in humans. The FPI model induces tissue displacements and progressive deformations of grey matter, cerebral edema, and intracranial hemorrhage through rapid injection of the pressurized fluid-filled piston into the epidural space (5, 15, 34). In particular, lateral models of FPI (LFPI) can induce both localized cortical contusions and diffuse neuronal injury across subcortical structures of the hippocampus and thalamus (35). The progressive cell death and DAI in LFPI models will persist up to 1-year post-injury. Furthermore, the LFPI-induced pathophysiological cascade will further progress across vulnerable subcortical regions of the striatum, medial septum and amygdala and cause subsequent cognitive impairments, movement disorders and neurobehavioral dysfunctions that last more than 1-year post injury, similar to the clinical trajectories of human TBI (10, 22, 36, 37).

Although these models have respective features which align with certain clinical contexts, the FPI and CCI models carry important limitations. Whereas moderate and severe cases of human TBI frequently carry skull fractures and substantial contusions across gyri, FPI and CCI models reproduce human TBI without clinically present skull fractures. In addition, clinical TBI is frequently characterized by chronic sleep disorders, vestibular deficits and severe headaches in patients following the injury. Extensive literature of the recent decade has elucidated that sleep–wake dysfunction is one of the most reproducible TBI model sequelae, with phenotypes of hypersomnolence, sleep fragmentation, and disrupted orexin signaling that reflect those seen in human patients. These studies emphasize that no model replicates the whole chronic symptom complex but that convergent animal and human data strongly implicate deranged sleep–wake circuitry as a mechanistic contributor to long-term morbidity after TBI (38–41). Furthermore, investigators have recapitulated isolated features of chronic TBI symptoms using a FPI mouse model to simulate mild TBI. These investigators found mice had difficulty in maintaining wakefulness (42). Stemper et al. (43) used a high-rate rotational acceleration model and showed sustained balance & anxiety-like changes that scaled with duration of acceleration.

Contemporary animal models of TBI, including FPI and CCI models, often omit secondary insults, which can complicate extrapolation to heterogeneous clinical populations (12, 44–46). Hence, prioritizing models that include secondary neurologic insults are likely to improve translational alignment. For instance, recent studies have devised randomized TBI + Hypoxemia models of diffuse brain injury in which elevated neuroinflammatory markers of TNFα, IL1-β and IL-6 corresponded to the reduced recovery of sensorimotor function 2 weeks post-injury (47–49). In addition, regions of concentrated axonal injury coincided with substantial astrocytosis and microglial activation (49). Such secondary insult experimental models are particularly promising for the clinical population as they are predictive models of treatment response and recovery rate immediately following the injury. Beyond recapitulating mechanical injury, these models have also been instrumental in deciphering the complex immunological landscape following TBI, providing insights into potential therapeutic targets, as explored in the next section. Table 2 elucidates on the emergent experimental models of TBI and their respective immunological insights.

**Table 2:** Experimental models of TBI and their immunological insights.

Model	Description	Immunological insights	Strengths	Limitations
Controlled cortical impact (CCI) (169)	Electromagnetic piston delivers cortical impact at controlled velocity and depth.	Induces acute cytokine release (TNF-α, IL-1β), BBB disruption, microglial priming, and delayed complement activation (170).	High reproducibility; well-suited for mechanistic and therapeutic studies.	Does not model diffuse injuries or secondary polytrauma seen in severe TBI.
Fluid percussion injury (FPI) (171)	Fluid pulse on intact dura induces mixed focal and diffuse injury.	Replicates systemic neuroinflammation, neutrophil infiltration, and prolonged astroglial activation (126, 172, 173).	Models diffuse injuries effectively induces persistent neuroinflammation similar to human TBI.	Less control over injury parameters; minimal replication of focal contusions.
Weight-drop model (174)	Free-falling object induces cortical contusions and hemorrhages.	Increases microglial reactivity, BBB permeability, and excitotoxicity (excessive glutamate release) (175, 176).	Simple and cost-effective; replicates severe cortical contusions.	Poor reproducibility; limited utility in modeling secondary systemic insults.
Penetrating ballistic-like brain injury (PBBI) (16)	High-velocity penetration of brain tissue mimics ballistic trauma.	Triggers chronic neuroinflammation and glial scarring (177–179).	Models severe inflammation and persistent immune dysregulation in penetrating injuries.	Highly invasive; difficult to standardize and ethically challenging.
TBI + secondary insults (180)	Combined TBI with hypoxia, hemorrhagic shock, or systemic inflammation.	Enhances IL-6, TNF-α, and MCP-1 signaling, worsening BBB permeability and neuroimmune dysfunction (52, 180).	Clinically relevant; mimics polytrauma conditions seen in severe TBI.	Complex methodologies; limited standardization across research groups.

## Immunological mechanism of traumatic brain injury

TBI initiates a multi-factorial cascade of immunological events which may serve as a basis for therapeutic target and intervention in future studies (50). Initial mechanical injury to the brain parenchyma leads to disruption of the BBB, which serves as an interface between the central nervous system and peripheral circulation (51). An impaired and permeable BBB is a pathological hallmark which precedes the immune cascade in TBI (52, 53). Immediately following injury, an inflammatory response is generated, which recruits glial cells (macrophages and astrocytes) to the site of injury, followed by peripheral immune cells, such as monocytes, natural killer cells, dendritic cells and T cells (47, 54). The activation of the immune system and the subsequent cascades are mediated by damage-associated molecular patterns (DAMPs), purinergic signaling, and the secretion of pro-inflammatory cytokines by glial cells and macrophages near the site of injury (55–57). During this time, the dysfunctional BBB also allows for continued trafficking of pro-inflammatory immune cells, leading to chronic neuroinflammation and cell death (58). Therefore, understanding the role of inflammation and its contribution to secondary injury in the brain following TBI could lead to the development of immune modulation therapies that improve long-term outlooks for TBI patients. Furthermore, the biphasic immune response in TBI mirrors the inflammatory dynamics of glioblastoma (59, 60), making TBI a valuable model for profiling GBM immunophenotypes. Insights into cytokine signaling, BBB disruption, and myeloid polarization in TBI may inform precision immunotherapy in GBM, in particular on the role of metabolic orchestrations that tumor cells utilize to instantiate immune evasions, many of which are abundantly present in post-TBI inflammation cascades (61, 62).

### Innate immune response

Microglia and astrocytes are the innate immune cell population in the CNS and play critical roles in neuroinflammation and repair following TBI. Microglia are known to disrupt the BBB when activated by NLRP3, a known pro-inflammatory marker (63). While the mechanism of this activation pathway is not fully elucidated, such process is thought to involve the recruitment of CXCR2-containing neutrophils by GDF-15 production (63). Additionally, astrocytes can exhibit neuroprotective and neurotoxic effects that are highly context dependent which allow for modulation of their behavior via inflammation-associated molecules. Astrocytes have impaired glutamate reuptake abilities following TBI which can lead to excitotoxicity following TBI (64, 65). This mechanism may be in part due to an imbalance of D-serine release between injured neurons and astrocytes at the site of injury (66). Continued excitotoxicity is linked to microglial activation and neuroinflammation via calmodulin-dependent protein kinase (CaMK), cAMP and extracellular signal-regulating kinase (ERK) pathways (67). Astrocytes are also implicated in maintaining the structural integrity of the BBB as they can release signaling molecules to affect BBB permeability. For instance, VEGF and APOE secretion by astrocytes increases leakiness of the BBB (68, 69). In contrast, sonic the hedgehog (SHH) genes or secretion of retinoic acid by astrocytes can reduce BBB permeability (70, 71). Transgenic mouse model without astrocytes showed greater cortical degeneration, demonstrating that astrocytes may play a protective role following TBI as their absence in TBI leads to neuronal degeneration and increased inflammation (72). Conversely, astrocyte activation following the circulation of inflammatory microRNAs was associated with pro-inflammatory state of astrocytes and contributes to secondary brain injury (73). Therefore, astrocytes demonstrate both neuroprotective or neurotoxic, which varies highly within the context of their microenvironment.

Populations of innate immune cells, such as neutrophils and monocytes, undergo proliferation in cervical and draining lymph nodes following TBI (74). The entry of these peripheral immune cells is permitted through the functionally disrupted BBB. M1 macrophages, activated by INF-*γ* and toll-like receptors (TLRs), cause neurotoxicity via inflammation induction whereas M2 macrophages promote axonal repair following TBI (75). Indeed, a high M1/M2 macrophage ratio has been reported to be detrimental to the reduction of inflammation in CNS injuries (76, 77). Furthermore, a study by Makinde et al. (78) found that circulating peripheral monocytes recruit neutrophils into the injured brain, propagating further breakdown of the BBB. In this model, mice were depleted of all peripheral monocytes, but retained microglia, demonstrating that abrogating peripheral monocyte and neutrophil infiltration following TBI could contribute to enhanced survival and cognitive recovery following TBI.

### Cytokine and chemokine signaling in TBI

Immediately after TBI (0–6 h), DAMPS released from necrotic neurons engage TLR2/4 on infiltrating neutrophils, upregulating TNF-*α* and IL-1β that promotes endothelial adhesion-molecule expression, matrix metalloproteinase release, and rapid phagocytic clearance of myelin and erythrocytic debris (79). However, persistence of a pro-inflammatory milieu beyond 72 h impedes oligodendrocyte progenitor maturation and synaptic pruning, suggesting phase-specific rather than blanket inhibition (80). Additionally, in the acute post-TBI period, levels of IL-1β are elevated, and neutralizing IL-1β with a monoclonal antibody has been shown to prevent secondary injury by inhibiting downstream microglial activation (81). Similarly, inhibiting TNF-*α* with 3,6-dithiothalidomide within 12 h post-TBI improves recovery outcomes in mouse models (82). IL-6, which can serve as a biomarker of inflammatory load in the central nervous system (CNS), is associated with a worse prognosis during the first year after TBI when elevated. IL-17, which plays a role in sustaining inflammation, is linked to secondary brain injury, as its inhibition by IL-23 abrogates neuronal apoptosis and improves neural function. Furthermore, transfection of astrocytes to produce and release IL-2 locally in the brain has demonstrated neuroprotective effects through the recruitment of T regulatory (Treg) cells (83). These findings collectively suggest that inflammation must be carefully modulated after TBI—both insufficient and excessive inflammation can hinder recovery, with prolonged or elevated inflammation leading to secondary injury.

### The adaptive immune response in TBI

T helper (Th) cell subsets play distinct roles in modulating neuroinflammation after traumatic brain injury (TBI), with Th1, Th2, and Th17 cells influencing the blood–brain barrier (BBB) and secondary brain injury through different mechanisms. Th1 cells produce pro-inflammatory cytokines (IFN-ɣ, IL-2 and IL-12) which can cause further harm. One mechanism by which Th1 cells increase neuroinflammation is by permeabilizing the BBB to allow greater uptake of leukocytes, and results in white matter injury (84). In contrast, Th2 presence is associated with anti-inflammatory cytokine release and neuroprotection in TBI (85). Specifically, Th2 inhibits the activation of microglia, and therefore serves to modulate the neuroinflammatory response following initial TBI (86). In addition, Th17 cells secrete IL-17, which is suspected to promote BBB disruption, increase CNS inflammation, and contribute to secondary brain injury through the IL-23, IL-17 axis (87).

Following TBI, B cells become activated and produce autoantibodies. Autoreactive CD19 + B cells increase in number in the spleen and cervical lymph nodes, with peak levels 8–10 days post-injury (88, 89). Autoantibodies are generated against brain-specific proteins, such as GFAP, myelin-associated glycoprotein (MAG) and myelin basic protein (MBP) (89, 90). Zhang et al. (90) found that elevated levels of anti-GFAP are negatively correlated with patient outcomes, demonstrating that Anti-GFAP may be monitored as a biomarker to correlate with long-term neurodegeneration post-TBI. A subset of B-cells, regulatory B-cells (Breg; CD1dhi CD5+), infiltrate perilesional cortex within 12–48 h, secrete IL-10 and IL-35, and suppress microglial NF-κB activation, thereby limiting reducing nearby axonal degeneration (91, 92). Additionally, persistent anti-MAG IgM autoantibodies are associated with elevated serum neurofilament light concentrations, which suggest an active neurodegeneration process (89). Furthermore, autoantibodies against MBP and phospholipids in CSF are correlated with increased injury severity and vascular complications (93). Notably, the presence of brain-derived antigens in lymphoid tissue was demonstrated to trigger an adaptive autoimmune response and may be associated with patient outcomes (94). Finally, the production of autoantibodies and its associated sequelae can last for many years after the injury and lead to ongoing neuroinflammation and neurodegeneration.

### Systemic immune dysregulation following TBI

Systemic inflammation following TBI is a contributor to secondary injury in the CNS. High levels of inflammation during the first 90 days post-injury generally lead to less favorable outcomes when recovery is evaluated at 6 and 12 months following TBI (95–97). The systemic inflammatory response is characterized by immune activity by both CNS and peripheral immune cells. As previously mentioned, microglia produce inflammatory molecules such as IL-1β, IL-6, IL-12, NO, or ROS (81, 91, 98–100). In addition to the release of these pro-inflammatory molecules, reactive microglia increase neuroinflammation by exhibiting phagocytic behavior on the astrocytic processes which extend to support the BBB, and thus increase BBB permeability (98). Microglia further sustain neuroinflammation through the recruitment of peripheral macrophages following TBI (99). In contrast, B cells demonstrate a neuroprotective role following TBI by downregulating the number of inflammatory processes occurring in the immune environment following TBI (100). This occurs through B cell secretion of IL-10 and IL-35 anti-inflammatory cytokines (91). Furthermore, B cells produce brain-derived neurotrophic factor (BDNF), which supports neuronal survival and recovery (101).

**Figure 1:**
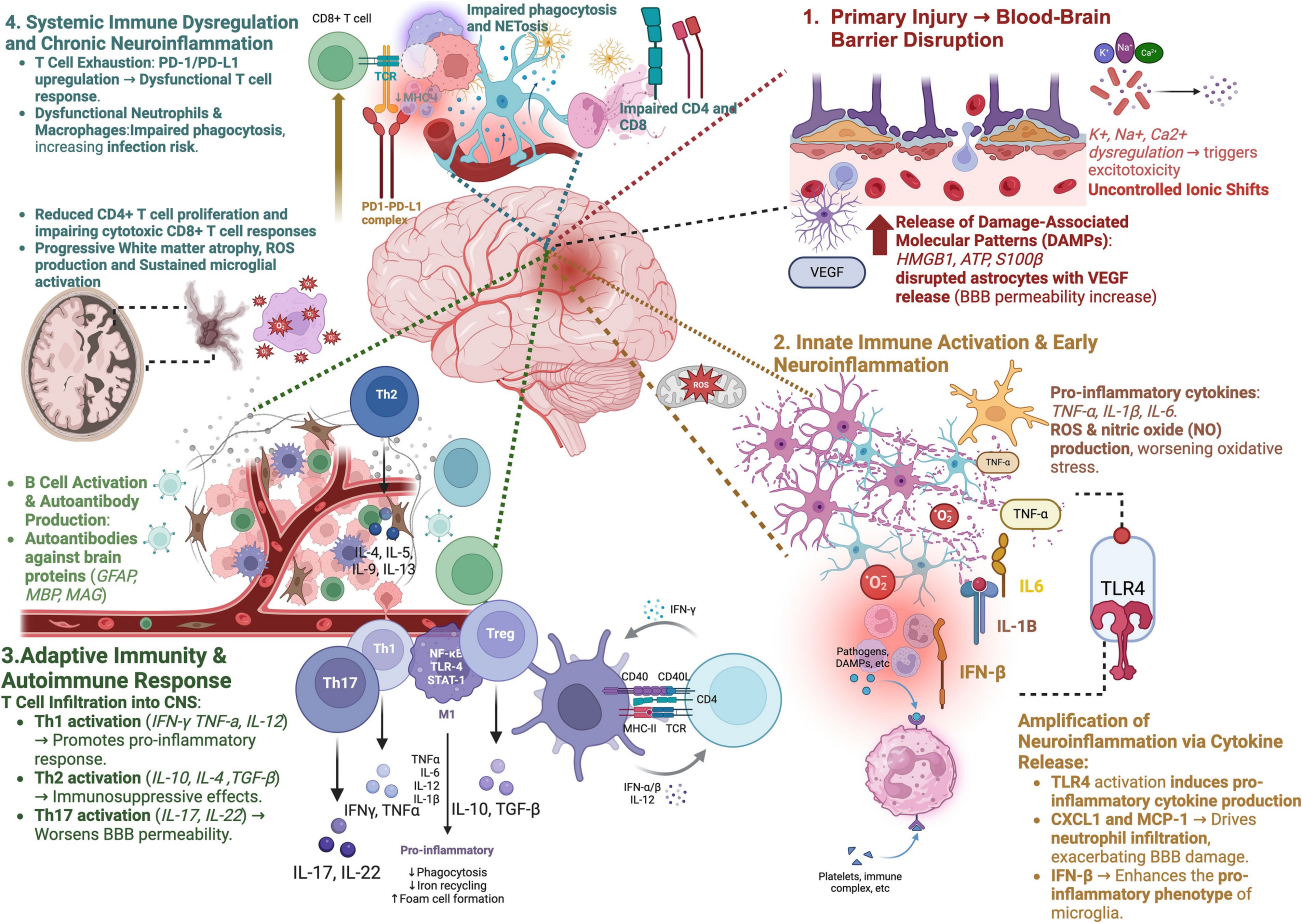
The immunological cascade of traumatic brain injury: from acute neuroinflammation to chronic systemic dysregulation and neurodegeneration. Traumatic brain injury (TBI) is characterized by a multistage immune response that ranges from acute neuroinflammation to systemic immune dysregulation and chronic neurodegeneration. Acute BBB breakdown permits damage-associated molecular patterns (DAMPs) to stimulate via TLR4 signaling the activation of microglial cells and the release of pro-inflammatory cytokines (TNF-*α*, IL-1β, IL-6, IFN-γ). This induces peripheral immune infiltration (neutrophils, monocytes, and T cells) and increases the permeability of BBB and the injury of neurons. Th1 and Th17 cells maintain inflammation, CD8 + T cells lead to the death of neurons (granzyme B, perforin) and B cells produce autoantibodies (anti-GFAP, anti-MBP, and anti-MAG), leading to development of autoimmunity of the CNS. Systemically, T cell exhaustion (PD-1/PD-L1) and diminished neutrophil phagocytosis in concert with peripheral inflammation increase responses to opportunistic infections. Chronically, long-term microglial priming, oxidative stress, and damage of blood–brain barrier (BBB) lead to white matter atrophy and synaptic loss, thereby increasing the risks for AD, PD, and chronic traumatic encephalopathy (CTE). This ongoing neuroimmune dysregulation calls for the development of directed immunotherapies to reduce long-term cognitive and functional decline. Figures were created using BioRender.com.

TBI impairs the function of key immune cells, namely macrophages, neutrophils, NK cells, and T cells, by disrupting immune responses and increasing susceptibility to infections. Notably, macrophages in patients with TBI have impaired phagocytic capabilities as well as impaired activation of NK cells, resulting in increased risk for infection (102, 103). Neutrophils are elevated in the first 48 h following TBI but are hyporesponsive and demonstrate a mitigated ability to phagocytose bacterial infections for up to several weeks following traumatic injury. This impaired immune response is suspected to be in response to neutrophil infiltration of the brain and subsequent preservation of brain tissue through downregulation of phagocytic behavior (104, 105). Additionally, the severity of NK cell depletion is correlated with severity of TBI and can persist for weeks following initial injury (106). Following TBI, the thymus shrinks, which correlates with the decrease in T cell circulation observed following TBI (107, 108). Th1 cells shift towards Th2 phenotype following TBI and the accompanying shift to Th2 cells predisposes patients to higher rates of infection (109). In concordance, PD-1 upregulation, a sign of immune cell exhaustion, is observed in T cells following TBI (110). Figure 1 recapitulates such immunological axis characteristic of traumatic brain injury.

### Immunology in experimental models of TBI

In order to better understand the underlying pathophysiology and immunological mechanisms of both primary and secondary insults following TBI, experimental models, such as CCI and FPI, have been utilized for their ability to recapitulate the immunological cascades following focal and diffuse TBI.

CCI has been shown to be an effective model for replicating the acute neuroinflammatory cascade following TBI (111, 112). In one study of mice undergoing CCI injury followed by biopsy, seven cytokines were measured, six of which showed significant elevation when compared to naïve controls (113). Following CCI injury, pro-inflammatory cytokines CXCL1, IL-1β, and IL-6 showed rapid elevation with peak expression at day +1. Three other pro-inflammatory cytokines, IL-12p70, IFN-*γ*, and IL-10, showed peak expression at day +3. Though not completely mirrored in humans, a number of pro-inflammatory cytokines are preserved in mice and have shown similar temporality and upregulation post-TBI. Elevated serum CXCL1 concentration <24 h post-TBI was positively correlated with TBI severity, and higher levels of CSF IL-6 in the acute phase post-TBI were associated with worse outcomes as measured by Glasgow Outcome Scale scores at 6 months following injury (114, 115). Another study utilizing cerebral microdialysis paired with arterial and jugular bulb plasma in six TBI patients showed that IL12-p70 and IL-10 peaked more than 3 days following injury, whereas IL-1β peaked less than 2 days post-injury (116). CCI has been shown to be an effective model for replicating the acute neuroinflammatory cascade following TBICCI has frequently been used to characterize acute neuroinflammatory cascades following TBI. In one study of mice undergoing CCI injury followed by biopsy, seven cytokines were measured, six of which showed significant elevation when compared to naïve controls (113). Following CCI injury, pro-inflammatory cytokines CXCL1, IL-1β, and IL-6 showed rapid elevation with peak expression at day +1. Three other pro-inflammatory cytokines, IL-12p70, IFN-*γ*, and IL-10, showed peak expression at day +3. Though not completely mirrored in humans, a number of pro-inflammatory cytokines are preserved in mice and have shown similar temporality and upregulation post-TBI. Elevated serum CXCL1 concentration <24 h post-TBI was positively correlated with TBI severity, and higher levels of CSF IL-6 in the acute phase post-TBI were associated with worse outcomes as measured by Glasgow Outcome Scale scores at 6 months following injury (114, 115). Another study utilizing cerebral microdialysis paired with arterial and jugular bulb plasma in six TBI patients showed that IL12-p70 and IL-10 peaked more than 3 days following injury, whereas IL-1β peaked less than 2 days post-injury (116).

The chronic inflammatory response following CCI extends well beyond the acute phase, demonstrating persistent neuroinflammation that mirrors human TBI pathology (117–120). In one study of CCI in moderate-level TBI mice, the chronic phase was characterized by progressive expansions of lesion volumes: 287, 309, and 483% increases at 5, 12, and 52 weeks post-TBI, respectively, along with microglial activation persisting up to 1 year post-TBI (121). These findings recapitulate those found in humans, where PET imaging of moderate to severe TBI survivors indicated increased microglial activation up to 17 years post-TBI (122). The extended inflammatory response represents a potential therapeutic window that extends well beyond the traditional acute treatment period, highlighting the importance of understanding and targeting chronic inflammation in TBI treatment strategies.

Despite its control and reproducibility, CCI may not adequately represent diffuse injuries (123). To better simulate these types of injuries, FPI is utilized, which is classified into two categories: midline FPI and lateral FPI. Midline FPI induces diffuse TBI with bilateral structural injury and inflammation while lateral FPI induces both diffuse and focal TBI. In the acute phase, FPI models have demonstrated significant neutrophil infiltration (124, 125). One study analyzed myeloperoxidase (MPO) activity, a specific marker of neutrophils, in rats which underwent trauma via FPI and saw that MPO concentration peaked at 24 h post-trauma (126). In severe TBI human patients, polymorphonuclear neutrophils (PMNs) have shown increased activation and decreased apoptosis, leading to levels up to three times that of controls for the first 24 h following injury (127). Furthermore, FPI models have shown upregulation of the pro-inflammatory cytokines IL-1β and TNF-*α* following TBI. In midline FPI, IL-1β mRNA was significantly upregulated at 24 h post-TBI, and TNF-α mRNA was significantly upregulated at 4 and 24 h post-TBI when compared to control mice (128). In brain tissue samples from 21 human TBI patients, both IL-1β and TNF-α were significantly overexpressed as well, suggesting that FPI captures immunologic responses that resemble clinical TBI in certain respects (129).

Several innate and adaptive pathways differ between rodents and humans. For instance, mice exclusively express the membrane-attack-complex inhibitor, CD59b, exclusively in their testis, as opposed to ubiquitous expression in humans, predisposing mice to heightened complement-mediated inflammation following TBI (130). Furthermore, mouse macrophage and dendritic cells express TLR11/12, absent in humans, which leads to heightened IFN- *γ* secretion (131). Given that this isoform of TLR is not functionally expressed by humans, this contributes an additional immune mechanism of M1 macrophage polarization that differs between mice and humans. In the adaptive compartment, C57BL/6 mice mount a rapid Vβ8.1/8.2 T-cell expansion driving IL-17 production, whereas human TCR repertoires show delayed, polyclonal activation (132). These discrepancies may underlie the failure of IL-17 blockade and complement inhibitors to replicate rodent efficacy in phase II trials.

Given the limitations of traditional models like CCI and FPI in replicating complex secondary injuries such as hypoxia, there has been a shift towards more sophisticated models. These advanced models are designed to include these secondary neurological insults, providing a better model which can recreate the complex realities of human TBI. The TBI + Hypoxia model, in particular, shows notable potential for translational application. A study by Davies and colleagues induced hypoxia in mice 1 day following TBI, and found this led to deficits in memory and learning along with increased astrocytic response when compared to TBI mice which did not undergo hypoxia (133). Other studies incorporating hypoxia as a secondary insult have shown elevated pro-inflammatory cytokines TNFα, IL1-*β* and IL-6 (134, 135). By incorporating secondary insults into these TBI models, the subsequent neuroinflammatory cascades more closely resemble human TBI patients, providing a promising direction for clinically translational TBI models. Given the critical role of neuroinflammation in secondary injury, emerging immunomodulatory therapies aim to mitigate these effects, offering new avenues for intervention. Table 3 provides an overview of fundamental mechanisms of resistance in emergent TBI therapeutics.

**Table 3:** Therapeutic strategies and their challenges.

Therapeutic domain	Current strategies	Emerging approaches	Challenges and limitations
Primary insults	Injury prevention (seatbelts, helmets).	Advanced neuroprotective gear incorporating rotational force dissipation (181).	Limited therapeutic intervention post-impact; relies on behavioral adherence.
Secondary insults	ICP monitoring, CPP optimization, hypothermia therapy.	BBB-permeable neuroprotective agents, biomarker-driven interventions (182).	Heterogeneity of TBI pathology complicates standardized treatment; failure of neuroprotective agents in large-scale trials.
Surgical interventions	Clot evacuation, decompressive craniectomy, CSF drainage.	Minimally invasive procedures, neuroimaging-guided interventions (183, 184).	Risk of infection, exacerbation of neuroinflammation, need for individualized treatment strategies.
Pharmacological interventions	Anticonvulsants, anticoagulants, anti-inflammatory drugs.	Targeted cytokine inhibition, nanoparticle-mediated drug delivery (185, 186).	Poor penetration across the BBB; systemic toxicity concerns.
Experimental models and research	FPI, CCI, TBI + polytrauma models.	Integration of multi-insult models, organoid-based TBI modeling (187, 188).	Limited translational success due to species differences; high experimental costs.

### Targeted immunologic therapy

TBI elicits a complex immunopathological cascade characterized by microglial activation, peripheral leukocyte recruitment, and elevated pro-inflammatory cytokines. Initial neuroprotective responses can transition to detrimental inflammation, exacerbating neuronal damage and impeding recovery. Advances in neuroimmunology have delineated the molecular and cellular mechanisms underpinning post-traumatic neuroinflammation, identifying targeted interventions such as cytokine antagonism, complement inhibition, and T cell modulation. These strategies aim to reduce secondary injury and enhance neurofunctional outcomes in TBI management.

Cytokine modulation has emerged as a potent therapeutic strategy for TBI, targeting the reduction of neuroinflammation and edema through the neutralization of pro-inflammatory cytokines. Among these, interleukin-1 receptor antagonists (IL-1ra) and TNF-*α* inhibitors have shown significant promise. Inhibition of NLRP3, an upstream inflammasome of IL-1β, in mice has been shown to attenuate neurological deficits in spatial learning and memory recovery after TBI (136, 137). Furthermore, brain edema and cortical lesion size were significantly reduced following inhibition of NLRP3 in mice. Anakinra, a recombinant form of the human IL-1ra, has been approved in humans for rheumatologic conditions and is now being trialed in humans for TBI (138). Another target for cytokine modulation is TNF-*α*, and anti-TNF-α agents, such as infliximab, are currently being explored as therapies for TBI, particularly for their ability to ameliorate endothelial dysfunction in the setting of TBI (139, 140).

Complement inhibition may serve as another potential therapy for TBI, preventing synaptic loss and neurotoxicity. Inhibition of C3 activation has been shown to reduce chronic neuroinflammation and neurodegeneration in mice following CCI (117). C5 deficient mice showed reduced brain lesion size when treated with C1-Inh and CR2-Crry and improved cognitive function following CCI when compared to control mice (141). Currently, anti-C5 antibodies such as eculizumab are being trialed for safety and efficacy in subarachnoid hemorrhage patients, but no trials have been conducted in the setting of patients with TBI (142).

T cell modulation has been seen as another potential therapeutic target for TBI patients. Various T cell subsets, namely V*γ*1 and Vγ4 γδ T cell subsets, play distinct roles in TBI pathophysiology. The former is responsible for activation of microglia and induction of neuroinflammation by secretion of IFN-γ and IL-17, and the latter dampens TBI and maintains microglial homeostasis through TGF-*β* secretion (143). CD8 + T cells have also been implicated in TBI pathophysiology, causing chronic neurological impairment through increased expression of GrB in activated CD8 + T cells, upregulating the GrB/perforin cytolytic pathway (144). Mice which were pharmacologically depleted of CD8 + T cells showed improved neurological outcomes following CCI.

Other emerging therapies which have shown promise but have not yet progressed to clinical trials include exosome therapy, immune checkpoint inhibitors, and precision immunology approaches. Exosome therapy works by utilizing engineered nanoparticles to deliver anti-inflammatory miRNAs or cytokine inhibitors. In one study of human adipose mesenchymal stem cell-derived exosomes (hADSC-ex) in TBI rats, the exosome therapy facilitated sensorimotor functional recovery, inhibited neuroinflammation, reduced neuronal apoptosis, and promoted hippocampal neurogenesis (145). Immune checkpoint inhibitors, namely the PD-1/PD-L1 pathway, have also been studied for their application in TBI. Following surgical brain injury in mice, administration of PD-L1, the ligand for PD-1, significantly reduced cerebral edema, and PD-L1 blockade exacerbated cell death *in vivo* (146). Furthermore, blockade of PD-L1 in post-TBI mice which underwent CCI led to increased cavity size of the injured cortex along with motor and emotion dysfunction, further highlighting that inhibiting T cells through PD-1 interaction may play a protective role in TBI (147). Given the possibility of overactivation of the immune system and subsequent non-specific inflammation, future studies involving immune checkpoint inhibition will need dose-escalation trials to satisfy safety requirements. While PD-1/PD-L1 modulation has been found to be potentially effective at reducing edema and inhibiting T-cell–mediated damage after TBI, the overall risk remains that of immune overdrive within the already inflamed and compromised environment of the CNS (146, 147). Excessive checkpoint blockade can potentially increase BBB disruption, amplify Th1/Th17-mediated cytokine cascades, and induce autoantibody formation against CNS antigens such as GFAP and MBP, thereby accelerating chronic neurodegeneration. Such concerns are further instantiated in GBM, where PD-1 blockade reveals CNS autoimmunity despite therapeutic response in patient populations (60, 148). Thus, new approaches must include biomarker-directed, time-limited checkpoint modulation, possibly in addition to adjuncts such as exosome delivery platforms or microbiome-directed approaches, to maximize the balance between protective immunity and pathologic inflammation.

Gut–brain axis modulation, a precision immunological approach, works by restoring microbiota through probiotics or fecal microbiota transplantation to reduce systemic inflammation and has been explored in mental health, inflammatory bowel disease, multiple sclerosis, and rheumatoid arthritis (148–150). Recent efforts have characterized the gut–brain axis as a therapeutic target for TBI as well (151). Table 4 provides an overview of emergent immunotherapeutic strategies in this venture.

**Table 4:** Immunological therapeutic targets in TBI.

Target	Mechanism of action	Therapeutic examples	Stage of development	Challenges
Cytokine modulation	Blocks pro-inflammatory cytokines to prevent neuroinflammation.	IL-1β antagonists (anakinra), TNF-α inhibitors (infliximab) (140, 185).	Preclinical and early-phase trials.	Systemic immunosuppression, narrow therapeutic window.
Microglial polarization	Shifts microglia from M1 (neurotoxic) to M2 (neuroprotective) phenotype.	PPAR-γ agonists (pioglitazone), TGF-β modulators (176, 189, 190).	Preclinical studies.	Risk of impairing microglial surveillance; limited *in vivo* specificity.
Complement Inhibition	Blocks C3a/C5a signaling to prevent neurotoxicity.	Anti-C5 antibodies (eculizumab) (142, 191).	Early-phase clinical trials.	BBB penetration challenges; increased infection risk.
Chemokine signaling blockade	Inhibits immune cell infiltration by targeting chemokine receptors.	CCR2 inhibitors, CXCR4 antagonists (192, 193).	Preclinical studies.	Risk of off-target immune suppression.
Exosome therapy	Delivers neuroprotective agents via engineered vesicles.	MSC-derived exosomes with ncRNAs modulate neuroinflammation and promote repair (194)	Preclinical research.	Efficiency of BBB crossing; manufacturing scalability.
Gut–brain axis modulation	Alters microbiota composition to regulate systemic inflammation.	Probiotics, fecal microbiota transplantation (FMT) (195, 196).	Early-stage research.	Individual variability in microbiota responses.

### Integration with clinical strategies

Integration of these immunological therapies with clinical strategies is essential for clinical relevance in TBI patients. Utilizing immunological biomarkers for patient stratification is one potential avenue by which we can create more targeted immunological therapies to treat TBI patients. Translationally relevant biomarkers must be consistent between CCI rodent models and human TBI patients (43, 123). One study showed correlational similarity between post-TBI rodent and humans for cytokines IL-1β, IL-6, G-CSF, CCL3, CCL5, and TNF-*α*, which were also associated with white matter integrity preservation (152). Targeting these specific cytokines may allow for more targeted immunological therapies in the future.

Future immune-based therapies must also complement existing TBI management strategies. Current TBI management focuses on prevention of secondary insults by avoiding hypotension and hypoxia through maintenance of cerebral perfusion pressure and cerebral blood flow. Continual monitoring of intracranial pressure and utilization of bedside maneuvers, hyperosmolar therapy, CSF drainage, pentobarbital coma, and decompressive craniectomy when appropriate are necessary as well (133, 153). Immune-based therapies are focused on reducing neuroinflammation and enhancing functional recovery. This strategy is suited for complementing current therapies focused on therapeutic interventional windows for secondary insults, limiting future complications such as risk of death and long-term neurological and cognitive damage.

## Future directions for research and clinical translation

Advancement in TBI research requires closing the translational gap between animal models and human disease. CCI and FPI remain of use but due to their poor ability to emulate diffuse injury, secondary insults, and chronic effects (sleep–wake disturbance, vestibular failure, and headache) predictability is compromised. Next-generation models are required to pair TBI with systemic stressors such as hypoxia or polytrauma, use humanized immune systems or brain organoids to address species differences, and standardized injury severities and readouts across laboratories. Essential endpoints to harmonize include blood–brain barrier integrity, cytokine and complement signaling (IL-1β, TNF-*α*, IL-6, IL-23/IL-17, C3/C5), immune cell phenotyping, and autoantibody tracking (anti-GFAP, MBP, MAG) that can be directly compared with human biospecimens.

Clinically, enriched longitudinal cohorts supplemented by biomarkers and imaging readouts would need to be developed in order to align immune signatures with recovery trajectories. This platform would permit patient stratification by biomarkers for adaptive trials instead of the one-size-fits-all approach that has unraveled previous therapeutic efforts. Near-term objectives include careful testing of cytokine and inflammasome blockade, complement inhibition, and T-cell modulation, alongside concomitant efforts to confirm pharmacodynamic biomarkers of target engagement. Optimal treatment windows of TBI inflammation must also be addressed by trials given the biphasic development of TBI inflammation.

Other than these main approaches, adjunctive therapies should be examined in well-characterized subgroups. Exosome therapy, modulation of the gut–brain axis, and orexin-targeted therapy for sleep disturbance due to TBI are only a few promising options. Multi-omics and spatial transcriptomics combined with clinical phenotyping will be needed in order to make the leap to precision immunotherapy, as the therapy will be adapted to the individual’s specific immune make-up. By combining preclinical rigor with biomarker-informed, mechanism-based clinical trials, the emergent research can shift towards precision therapies that substantially improve long-term neurological and cognitive outcomes.

## Concluding remarks

Recent advances in experimental TBI models have enabled more accurate replication of human secondary injury cascades, including dysregulated cerebral blood flow, neuroinflammation, and diffuse axonal injury (154). Unlike earlier models, which emphasized focal insults, new paradigms emphasize the systemic and dynamic nature of secondary damage. Multifactorial models, including the addition of hypotension, radiation, or polytrauma, more closely replicate clinical presentation and may more validly predict treatment response. Immunopathologically, TBI progresses in a biphasic manner: an acute microglial activation, neutrophil invasion, and DAMP-mediated breakdown of the BBB pro-inflammatory process, and a chronic maladaptive immunity subsequently characterized by persistent M1 macrophage activation, oxidative stress, and excitotoxicity. Adaptive immune processes such as Th1/Th17-mediated damage and Th2/Treg-mediated modulation also determine long-term outcome, while autoantibodies to CNS antigens such as GFAP and MBP contribute to progressive neurodegeneration.

Moving forward, precision-targeted immunomodulation offers a compelling therapeutic avenue. IL-1β, TNF-α, and C5a inhibitors have all shown a potential to reduce secondary injury, and novel approaches, including exosome-mediated cytokine delivery and microbiota modulation, are emerging ventures. The introduction of immunophenotyping and biomarker-based stratification into the clinic will be instrumental in advancing beyond generalized neuroprotection. Lastly, the integration of multi-omics and spatial transcriptomics with patient-specific immune profiling has the potential to shift the field toward personalized, mechanism-driven therapies that more effectively address the heterogeneity of human TBI.
